# Nonselective and A2a-Selective Inhibition of Adenosine Receptors Modulates Renal Perfusion and Excretion Depending on the Duration of Streptozotocin-Induced Diabetes in Rats

**DOI:** 10.3390/ph16050732

**Published:** 2023-05-11

**Authors:** Joanna Dorota Sitek, Marta Kuczeriszka, Agnieszka Walkowska, Elżbieta Kompanowska-Jezierska, Leszek Dobrowolski

**Affiliations:** Department of Renal and Body Fluid Physiology, Mossakowski Medical Research Institute, Polish Academy of Sciences, 02-106 Warsaw, Poland

**Keywords:** adenosine receptors, hyperglycaemia, hydrogen peroxide, nitric oxide, renal blood flow, renal excretion, streptozotocin

## Abstract

Long-lasting hyperglycaemia may alter the role of adenosine-dependent receptors (P1R) in the control of kidney function. We investigated how P1R activity affects renal circulation and excretion in diabetic (DM) and normoglycaemic (NG) rats; the receptors’ interactions with bioavailable NO and H_2_O_2_ were also explored. The effects of adenosine deaminase (ADA, nonselective P1R inhibitor) and P1A2a-R-selective antagonist (CSC) were examined in anaesthetised rats, both after short-lasting (2-weeks, DM-14) and established (8-weeks, DM-60) streptozotocin-induced hyperglycaemia, and in normoglycaemic age-matched animals (NG-14, NG-60, respectively). The arterial blood pressure, perfusion of the whole kidney and its regions (cortex, outer-, and inner medulla), and renal excretion were determined, along with the in situ renal tissue NO and H_2_O_2_ signals (selective electrodes). The ADA treatment helped to assess the P1R-dependent difference in intrarenal baseline vascular tone (vasodilation in DM and vasoconstriction in NG rats), with the difference being more pronounced between DM-60 and NG-60 animals. The CSC treatment showed that in DM-60 rats, A2aR-dependent vasodilator tone was modified differently in individual kidney zones. Renal excretion studies after the ADA and CSC treatments showed that the balance of the opposing effects of A2aRs and other P1Rs on tubular transport, seen in the initial phase, was lost in established hyperglycaemia. Regardless of the duration of the diabetes, we observed a tonic effect of A2aR activity on NO bioavailability. Dissimilarly, the involvement of P1R in tissue production of H_2_O_2_, observed in normoglycaemia, decreased. Our functional study provides new information on the changing interaction of adenosine in the kidney, as well as its receptors and NO and H_2_O_2_, in the course of streptozotocin diabetes.

## 1. Introduction

Long-term elevated blood glucose levels (hyperglycaemia, DM) cause vascular impairment and dysfunction in many organs, including the kidney. It is postulated that the development of diabetes-associated renal dysfunction may be the result of disorders of local paracrine agents synthesis and/or activity; one of them is the adenosine (Ado) system (including catabolic enzymes, nucleoside transporters, and receptors) [1,2]. The dysfunction may also be associated with nitric oxide (NO) tissue bioavailability alterations, and also the increased production of reactive oxygen radicals (ROS) including H_2_O_2_ [1,3,4,5].

Pharmacologically, Ado-dependent receptors (P1R) belong to the G protein-coupled receptor family, and they are named A1, A2a, A2b, and A3 [6]. Ado has vasodilatory activity on the vascular beds of most tissues. In the renal vasculature, Ado causes either constriction or dilation, depending on which receptor is predominantly stimulated, A1 or A2 (A1R, A2R), respectively [7]. The vasodilatory effect, mediated mainly by NO, may be especially important in the renal medulla, which operates under relative hypoxia and is susceptible to ischaemic damage. On the other hand, oxidative stress affects disruption of this environmental equilibrium and leads to medullary injury [8,9]. Therefore, the final biological effects of endogenous Ado depend on which P1R subtype prevails in the area.

The density of Ado-dependent receptor subtypes differs between the renal zones cortex and medulla. Most often, in the cortex, compared to the medulla, a higher A1R/A2R ratio has been described, whereas an opposite pattern for A2R vs. A1R expression was reported [10]. Some data indicated that the abundance of Ado receptor subtypes is altered in pathological conditions, e.g., in diabetes mellitus (DM) [1,11]. The results of receptor expression studies in the renal cortex and medulla of DM animals were discrepant [12,13], and the functional importance of the findings is not obvious [2].

Remarkably, activation of P1R depend also on the actual availability and distribution of the enzymes of the adenosine system. Adenosine deaminase (ADA) can rapidly alter the extracellular Ado content and the activity of P1R subtypes and, consequently, the ultimate effect in a particular kidney zone [14].

Renal dysfunction in DM has been linked to the dysregulation of local NO synthesis and ROS production [15,16,17]. Depending on the stage of the disease, NO bioavailability markedly differs: it was found to increase initially, then reduce in the advanced stage [18,19,20]. Nevertheless, in rats it was shown with intact NO synthesis measurements using NO sensitive microelectrodes, that regardless of the level of glycaemia, a nonselective Ado receptor blockade with theophylline increased kidney tissue NO, especially in the medulla. Apparently, theophylline may in some way abolish the NO-inhibitory action of Ado receptors, likely of the A1 subtype [21]. The study suggested the prevalence of A2 over A1 influences renal vascular tone and tubular transport in diabetes [21]. On the other hand, the renal medulla is probably the target for early diabetes-related oxidative stress-induced damage, and H_2_O_2_ mediates renal medullary dysfunction in streptozotocin-induced (STZ) diabetes [22]. An increase in medullary H_2_O_2_ generation in diabetes may be responsible for hypoxic dysfunction of the medulla, secondary to a decrease in medullary perfusion, and also via enhancement of sodium reabsorption; both processes contribute to local tissue oxygen tension [22]. In addition, there is evidence that A2aR activation increases ROS generation; it may also activate eNOS, leading to enhanced NO generation, which may exacerbate the deleterious effects of ROS and oxidative stress [23].

Considering these complex unresolved issues, we decided to explore interactions of the Ado systems with NO and ROS in the control of blood circulation, both in the kidney and in the whole body, as well renal excretion. We analysed these interactions between normo- and hyperglycaemic animals. Given the availability of all the detailed information on the functional characteristics of Ado receptors, as well NO and H_2_O_2_ interrelations and actions in different states, we supposed that it is meaningful to evaluate, in a whole animal study, the Ado system’s regulatory role, and explore its operative links with NO and H_2_O_2._ In the present study, we compared the data from STZ-treated rats and from age-matched rats treated with a vehicle (controls); the STZ model is well-related to human type 1 diabetes. The effects on the renal haemodynamics and excretion of ADA, an enzyme that reduces the level of adenosine (endogenous agonist of A1, A2, and A3 receptors) and the effects of A2aR selective antagonist, were determined in anaesthetised NG and DM rats with short- or long-lasting hyperglycaemia. This was carried out in view of the lack of research on possible alterations in the roles of these receptors in the initial versus established phases of experimental diabetes. It is noteworthy that purinergic receptors were often proposed as a possible therapeutic target for several pathologies, including complications of diabetes mellitus [23,24]. Importantly, the studies included amperometric measurements of NO and H_2_O_2_ signals in the renal medullary tissue in situ.

## 2. Results

### 2.1. Chronic Effects of Streptozotocin Pretreatment on Metabolic and Renal Excretion Parameters

**Figure 1:**
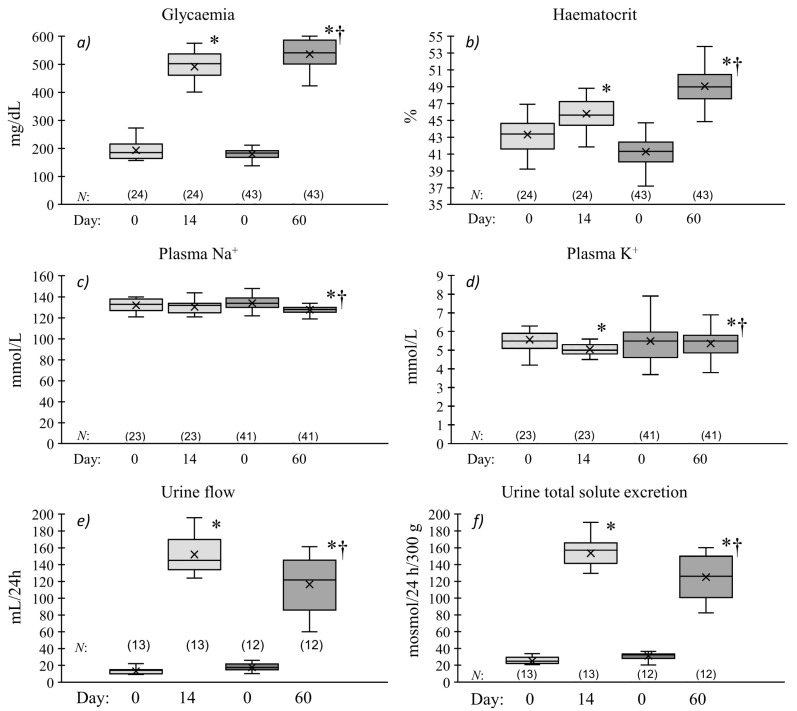
Blood, plasma (**a**–**d**), and renal excretion parameters (**e**,**f**) for Tac:Cmd:SD rats before (Day 0) and 14 or 60 days after streptozotocin administration. Measurements in tail vein samples and in metabolic cages were performed before *i.p.* streptozotocin (STZ) injection (Day: 0), and 3 days before acute experiments performed on day 14 (light grey boxes) or 60 (dark grey boxes) after STZ administration. The value distribution is shown as a box-and-whisker plot (the low and upper boundary of the box indicate 25th and 75th percentiles, respectively; the median is shown as the line inside the box; the mean value is marked with “×”. Whiskers drawn below and above the box show the 10th and 90th percentiles, respectively); N values are indicated in square brackets; *p =* 0.05 was taken as borderline significance: * different vs. the preceding day 0; † different vs. day 14.

The main differences between the data pooled from all of the rat groups, separately those with short- and long-term hyperglycaemia (day 14 and day 60, respectively) detected at the last metabolic cage observation are summarised in Figure 1. Compared with day “0”, the blood glucose level (glycaemia), haematocrit (Hct) and 24 h excretion parameters (urine flow—V and total solute excretion—UosmV) were elevated in both groups of animals. However, hyperglycaemia and Hct changes were significantly more pronounced in the rats with long- than with short-term diabetes (Figure 1a,b). This was in contrast to the changes observed in renal excretion that were more distinct in the latter group (Figure 1e,f). With the exception of plasma Na^+^, no change from the day “0” was seen in the day 14 rats (132 ± 1 vs. 131 ± 1 mmol/L), while on day 60 it decreased significantly (128 ± 1 vs. 134 ± 1 mmol/L) (Figure 1c). The plasma K^+^ level was reduced in both diabetic groups; however, this was more pronounced in rats with short-lasting hyperglycaemia.

The data for other parameters measured in normoglycaemic and hyperglycaemic rats at the beginning and the end of chronic observation are collected in Supplementary Materials Table S1. As expected, the body weight gain was lower in STZ-induced diabetic than in normoglycaemic age-matched rats. Likewise, the Hct and plasma osmolality (Posm) were also higher in DM than in NG rats, which indicates body dehydration in the former group of animals, despite free access to water. In addition, the higher Hct in the DM-60 rats in comparison with the DM-14 rats was associated with lower daily water intake (WI). Interestingly, a parallel change pattern in Hct, Posm, and WI was seen for the longer observation period in normoglycaemic (NG-60) rats. The renal excretory parameters on the last metabolic cage measurement were lower in NG than in DM animals (compared with the initial day “0” observations). On the whole, it was seen that the age and duration of hyperglycaemia significantly affected most of the metabolic and renal parameters.

### 2.2. Short- (14 Days) and Long-Term (60 Days) Effects of STZ-Induced Hyperglycaemia on Blood Pressure and Renal Haemodynamics and Excretion in Anaesthetised Rats

Following 14 or 60 days of observation in DM and NG rats within our chronic studies, the animals were anaesthetised to perform acute experiments. The baseline data for the acute experiments are collected in Table 1.

The mean arterial blood pressure (MABP) did not differ between short-term hyper- vs. normoglycaemic rats (DM-14 vs. NG-14), whereas it was significantly lower by about 10 mmHg in DM-60 rats compared to the NG-60 group. The total renal blood flow (RBF) only tended to be lower in the DM-14 compared to the NG-14 rats (difference NS), and was significantly lower in DM-60 compared to NG-60 animals. While the calculated resistance of the vasculature in the whole kidney (RVR) increased in both diabetic groups compared with the normoglycaemic groups, only the difference between DM-60 and NG-60 reached a statistically significant level. Noteworthily, the superficial cortical blood flow (CBF) did not vary between the DM-14 and NG-14 rats, but in a similar manner to whole kidney blood flow (RBF), it was visibly lower in the rats with long-term diabetes compared with their non-diabetic counterparts. 

The renal excretion parameters V, UosmV, and sodium excretion (UNaV), were higher in DM compared with NG rats, whereas the urine osmolality (Uosm) did not differ. Likewise, there was no difference between the DM-14 vs. NG-14 and the DM-60 vs. NG-60 groups (Table 1). On the other hand, potassium excretion (UKV) was lower in short-term DM compared with the corresponding NG group.

### 2.3. ADA Effects on MABP and Total and Regional Blood Circulation in Kidneys of NG and DM Rats

**Figure 2:**
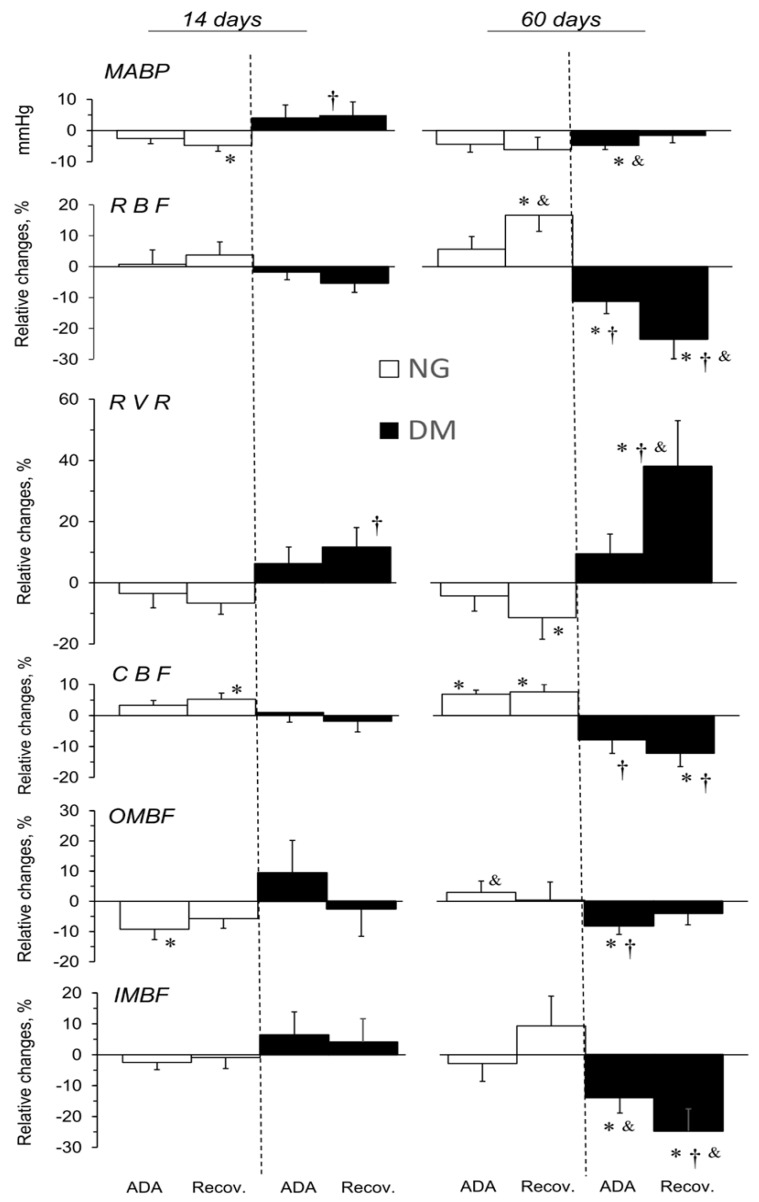
Effects of adenosine deaminase (ADA) on systemic and renal haemodynamics in normo- and hyperglycaemic rats (NG and DM, respectively) determined from acute experiments performed 14 or 60 days after streptozotocin or vehicle injection. The maximal stable responses induced by ADA and the values measured at the end of recovery period are shown as absolute MABP changes, and as percent mean differences (mean ± SEM) from the baseline values for other parameters. MABP, RVR and RBF, CBF, OMBF, IMBF—mean arterial blood pressure, renal vascular resistance and whole kidney, cortical, outer, and inner medullary blood flow, respectively. *p* = 0.05 was set as borderline significance: * different vs. the preceding baseline value, † different vs. the change in the corresponding NG group, & different vs. the change in the 14-day group.

The effects of ADA administered in acute experiments conducted 14 or 60 days after STZ or vehicle injection, expressed as relative differences from the pre-treatment value, are shown in Figure 2. There was a post-ADA decrease in MABP by about 5 mmHg, in both the NG-14 and NG-60 rats; however, the change was not significant in the NG-60 rats. In STZ-treated rats, MABP alterations appeared to depend on the duration of the hyperglycaemia.

ADA did not significantly alter RBF in the DM-14 and NG-14 rats; a similar pattern was seen in the corresponding control groups (see Supplementary Materials Figure S1). With long-term observation, distinct post-ADA alterations were seen: RBF significantly increased in NG-60 but decreased in DM-60 rats; evidently, the response depended on the glycaemia level. Any changes in the time control rats were not significant (Figure S1).

Simultaneously with the RBF changes, ADA increased the RVR in DM and lowered it in NG rats. Again, in the 14-day groups, the changing pattern was not different from that in the vehicle-treated groups (Figure S1); in NG-60 and DM-60 rats, the inverse post-ADA changes in NG and DM rats were very pronounced.

In DM-60 rats, only ADA clearly decreased cortical blood flow (CBF), whereas a slight increase was seen in the NG-14 and NG-60 groups. Noteworthily, no change in CBF in ADA-treated DM-14 rats was in contrast to a decrease observed in the vehicle-injected group (see Supplementary Materials Figure S1).

The effects of ADA on outer medullary blood flow (OMBF) were modified by hyperglycaemia, and depended on its duration; an apparent increase (NS) seen in DM-14 contrasted with a clear decrease in DM-60 rats. In the NG-14 and NG-60 groups, a decrease and no substantial effect were seen, respectively (Figure 2).

While there was no difference in ADA effects on inner medullary blood flow (IMBF) between the NG-14 and NG-60 rats, a pronounced significant decrease was seen in the DM-60 group. Notably, the post-ADA tendency to increase IMBF in the DM-14 and NG-60 rats was in contrast to the decrease shown in the corresponding time control group (Supplementary Materials Figure S1).

In general, no significant or consistent alterations were observed in MABP or renal perfusion in normo- and hyperglycaemic rats during ADA vehicle infusion, apart from the mentioned decreases in the RBF, CBF, and IMBF in rats with short-term diabetes, and of IMBF in long-term diabetes (Figure S1).

### 2.4. ADA Effects on Excretory Function in Kidneys of NG and DM Rats

**Figure 3:**
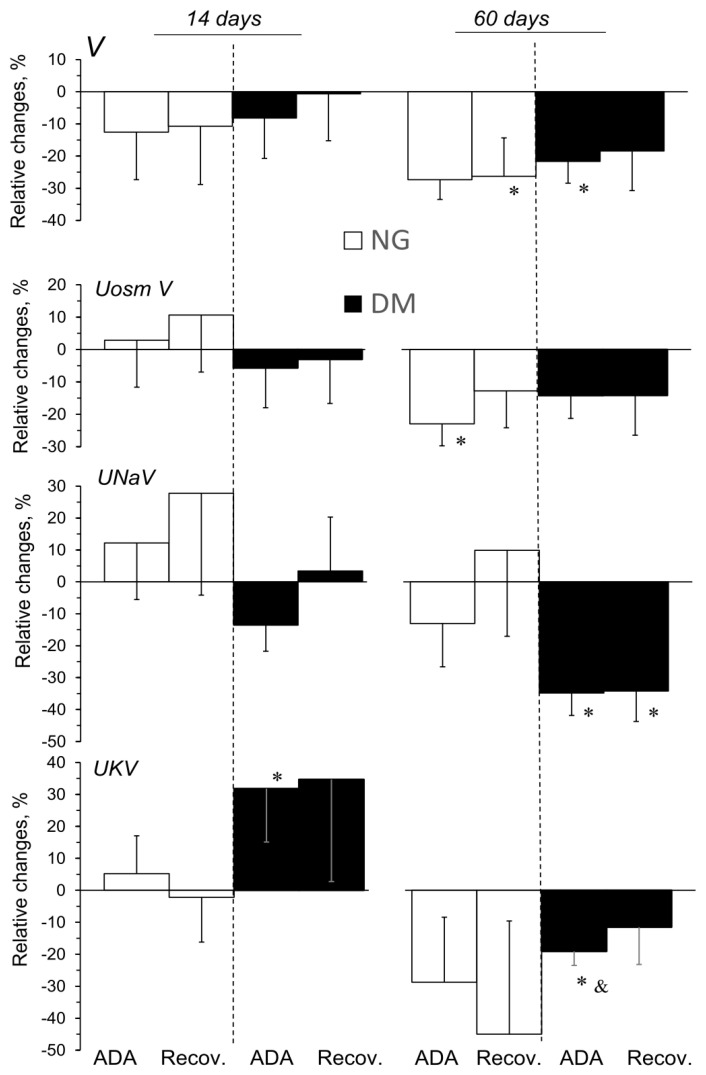
Effects of adenosine deaminase (ADA) on excretory function of the kidneys in normo- and hyperglycaemic rats (NG and DM, respectively) measured in acute experiments performed 14 or 60 days after streptozotocin or vehicle injection. The maximal stable responses induced by ADA are provided, as well as the values measured in the last period of recovery after ADA infusion, and are shown as percent mean differences (mean ± SEM) from the preceding baseline value. V and UosmV, UNaV, and UKV—urine flow and renal excretion of total solute, sodium, and potassium, respectively. *p* = 0.05 was set as borderline significance: * different vs. the preceding baseline value, & different vs. the change in the 14-day group.

The effects of ADA administered in acute experiments, expressed as relative changes from the pre-treatment value, are shown in Figure 3. The effects of ADA on some renal excretion parameters differed slightly depending on the duration of the hyperglycaemia (Figure 3), whereas no consistent changes were shown in NG and DM rats when ADA solvent was administered.

ADA caused a significant decrease in V for both the NG-60 and DM-60 groups, whereas UosmV decreased only in the NG-60 group. UNaV appeared to not consistently be altered by ADA in NG rats; however, in DM-60 rats it significantly decreased. Interestingly, depending on the duration of the hyperglycaemia, ADA altered UKV in the opposite direction: an increase or a decrease was seen in DM-14 and DM-60 animals, respectively.

### 2.5. CSC Effects on MABP, and Total and Regional Blood Circulation in Kidneys of NG and DM Rats

**Figure 4:**
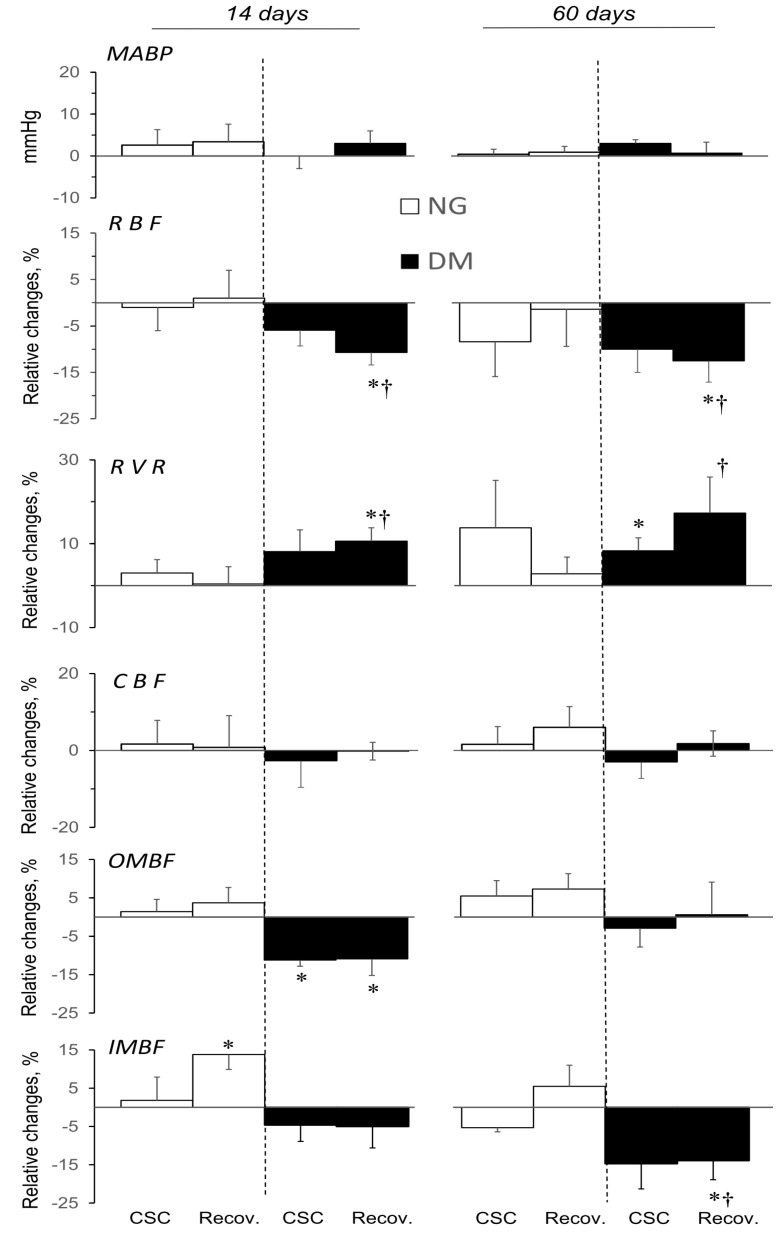
Effects of CSC (A2a receptor antagonist) on systemic and renal haemodynamics in normo- and hyperglycaemic rats (NG and DM, respectively), determined 14 or 60 days after streptozotocin or vehicle injection. The maximal stable responses induced by CSC and the values measured at the end of the recovery period are shown as absolute MABP changes and as percent mean differences (mean ± SEM) from baseline values for other parameters. MABP, RVR and RBF: CBF, OMBF, and IMBF—mean arterial blood pressure, renal vascular resistance and blood flow: whole kidney, cortical, outer, and inner medullary, respectively. *p* = 0.05 was set as borderline significance: * different vs. the preceding baseline value; † different vs. the change in the corresponding NG group.

The effects of CSC (A2a receptor antagonist) administered in acute experiments conducted 14 or 60 days after STZ or vehicle injection, expressed as relative changes from the pre-treatment value, are shown in Figure 4. CSC administration did not alter MABP in STZ-treated hyperglycaemic rats (DM, short- and long-term protocols), nor did MABP change in NG rats, irrespective of the age of the animals. Heart rate (HR) did not alter during CSC infusion; the exception was a decrease in DM-14 rats from the baseline of 350 ± 10 to the end value of 327 ± 5 beats/min (*p* < 0.05).

In animals with hyperglycaemia, irrespective of its duration, CSC induced a sustained and significant decrease in RBF (Figure 4). Dissimilarly, in normoglycaemic rats (NG-14 or NG-60), CSC did not cause any significant changes.

Consistently with the post-CSC decrease in RBF at stable MABP in diabetic rats, the calculated RVR increased substantially in a sustained manner.

For CBF, no changes were observed, similarly in DM and NG rats. CSC appeared to decrease OMBF in NG-14 rats, and reduced it significantly in their DM counterparts, in parallel with the decrease in RBF. However, in DM-60 rats, the RBF decrease was not associated with any change in OMBF.

IMBF was significantly altered in DM-60 rats only: a distinct sustained post-CSC decrease was seen. Interestingly, in normoglycaemia, the renal perfusion was significantly elevated by CSC only in the inner medulla.

No substantial, consistent, or meaningful changes in MABP or renal haemodynamics were observed during the infusion of CSC solvent (Figure S2).

### 2.6. CSC Effects on Excretory Function in Kidneys of NG and DM Rats

**Figure 5:**
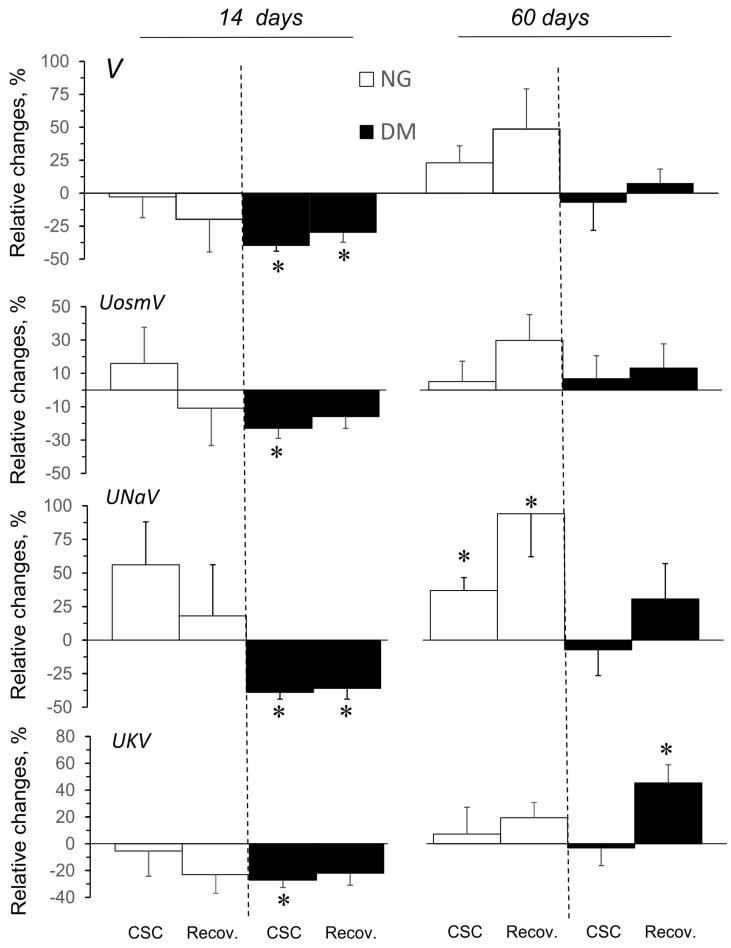
Effects of CSC (A2a receptor antagonist) on excretory function of the kidneys in normo- and hyperglycaemic rats (NG and DM, respectively), determined 14 or 60 days after streptozotocin or vehicle injection. The maximal stable CSC induced responses and the values observed in the last period of recovery after CSC infusion withdrawal are shown as mean differences from baseline values (mean ± SEM). V and UosmV, UNaV, and UKV—urine flow and renal excretion of total solute, sodium, and potassium, respectively. *p* = 0.05 was set as borderline significance: * different vs. the preceding baseline value.

The data on V, UosmV, UNaV, and UKV changes caused by CSC are summarised in Figure 5. Similarly to the effects of CSC on the haemodynamics (Figure 4), alterations in the excretory parameters were more pronounced in the kidneys of hyper- compared to normoglycaemic rats (Figure 5). The V, UosmV, UNaV, and UKV all decreased after CSC infusion in the DM-14 group; meanwhile, in the DM-60 groups, no significant alterations in renal excretion were shown; an exception was the post-CSC increase observed in UKV (45 ± 14%) in hyperglycaemic and UNaV (94 ± 32%) in normoglycaemic rats (Figure 4). Notably, the post-CSC increase in UKV was in contrast to the decrease shown in solvent-treated DM-60 animals. On the other hand, the increase in UNaV seen in NG-60 group resembled the effect seen in the corresponding time control experiment.

While CSC did not distinctly affect renal excretion parameters in NG-14 rats, they increased during the drug solvent infusion. Moreover, in DM-14 rats, UosmV and UNaV appeared to increase (NS) after solvent infusion.

### 2.7. ADA Effects on Tissue NO and H_2_O_2_ Signals in Kidneys of NG and DM Rats

**Figure 6:**
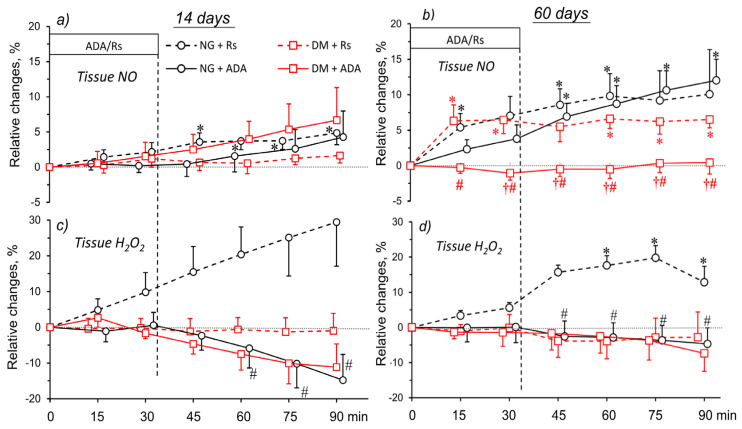
Effects of adenosine deaminase (ADA) or its solvent (Rs) on NO and H_2_O_2_ signals in situ in the kidney medulla of normo- and hyperglycaemic rats (NG and DM, respectively), measured in acute experiments 14 (**a**,**c**) or 60 days (**b**,**d**) after streptozotocin or vehicle injection. The time course of changes induced by ADA or Rs (Ringer solution), observed after drug or solvent withdrawal. The data are expressed as mean differences (mean ± SEM) from baseline, *N* = 5–8. *p* = 0.05 was set as borderline significance: * different vs. the preceding baseline value, † different vs. the change in the corresponding NG group, and # different vs. the change in solvent-infused rats.

Figure 6 collects the data on the effects of ADA or solvent (Ringer solution, Rs) on renal tissue NO and H_2_O_2_ in groups with 14 and 60 days’ hyperglycaemia, and in normoglycaemia. Before the start of ADA or Rs infusion, NO and H_2_O_2_ fluctuations were negligible, and are not shown in the figure. In the 14-day groups, NO fluctuations showed no difference between NG and DM animals, irrespective of the infusion (ADA or Rs) (Figure 6a). This was not the case with the 60-day groups, where those with hyperglycaemia showed stable tissue NO throughout the experiment, whereas a progressive increase was seen that finally became significant in all of the other groups (Figure 6b).

With short-term observation (Figure 6c), tissue H_2_O_2_ was very stable in DM rats receiving ADA solvent, in contrast to a decrease in ADA-treated NG and DM rats. With long-term observation, again, a significant difference between ADA-treated and solvent-infused NG groups was shown. In DM rats, no difference in tissue H_2_O_2_ changes were shown between ADA- and solvent-infused rats (Figure 6d).

Noteworthily, with short-term observation (Figure 6a,c), both in NG and DM rats receiving ADA tissue, NO and H_2_O_2_ altered in opposite directions: a minor increase in the former and a distinct decrease in the latter were seen. With long-term observation, this relationship was more pronounced in NG rats, and disappeared in DM rats.

### 2.8. CSC Effects on Tissue NO and H_2_O_2_ Signal in Kidneys of NG and DM Rats

**Figure 7:**
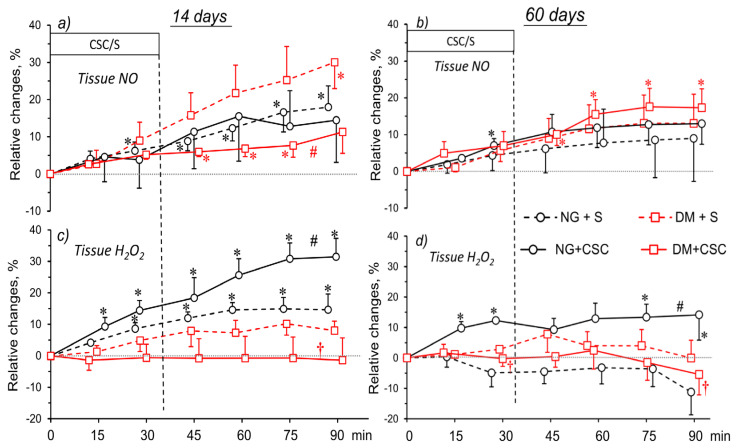
Effects of A2a receptor antagonist (CSC) or its solvent (S) on in situ NO and H_2_0_2_ signals in the kidney medulla of normo- and hyperglycaemic rats (NG and DM, respectively), measured in acute experiments 14 (**a**,**c**) or 60 days (**b**,**d**) after streptozotocin or vehicle injection. The time course of changes induced by CSC or S (8% DMSO in saline solution), observed after drug or solvent withdrawal, are shown as mean differences (mean ± SEM) from baseline. *N* = 5–8. *p* = 0.05 was set as borderline significance: * different vs. the preceding baseline value, † different vs. the change in the corresponding NG group, and # different vs. the change in solvent-infused rats.

In Figure 7, the data summarise the effects of CSC or its solvent (S) on renal tissue NO and H_2_O_2_, as related to the animals’ age or duration of hyperglycaemia. Before the administration of CSC or its solvent, the NO and H_2_O_2_ alterations were negligible and omitted from the figure.

In NG-14 rats, there was no difference in tissue NO fluctuations after CSC or solvent treatment; meanwhile, in DM-14 tissue, NO differed between CSC and S-infused groups at the end of the experiment (Figure 7a). Likewise, in the NG-60 group, no difference was seen between tissue NO alteration after CSC or S. However, in diabetic animals, a significant increase in tissue NO was seen after CSC; however, this response was no different from the change seen after the solvent (Figure 7b).

Interestingly, after the CSC tissue H_2_O_2_ increased 30% from baseline in NG-14 rats, the change was clearly different from alterations observed in other groups (Figure 7c). Remarkably, in NG-60 tissue, H_2_O_2_ was also elevated by CSC, but the increase (by 10%) was smaller than that in NG-14 tissue (Figure 7d). At the end of the experiment, the changes observed were different from fluctuations in the solvent-infused group, as well as in those seen in diabetic rats (Figure 7d).

## 3. Discussion

(1) The present study compared the role of endogenous adenosine and its interaction with NO and H_2_O_2_ in controlling renal and systemic circulation and renal excretion between rats without diabetes and those with streptozotocin-induced diabetes.
(2) The study was extended to compare these relationships between short- and long-term diabetic and age-matched normoglycaemic rats.
(3) We postulated that in the kidney, the role of endogenous adenosine on circulation and excretion differs, depending on the actual duration of the experimental diabetes.

### 3.1. Chronic Observations

We found that in male Sprague–Dawley rats, STZ-induced hyperglycaemia was sustained 2 weeks after injection (short-term studies); however, the blood glucose level was slightly but significantly elevated 8 weeks after STZ injection (long-term studies). Remarkably, group body weight (BWt) gain was seen in neither group, despite hyperphagia. This was in contrast to normoglycaemic rats (Figure 1). This accords well with recent [25] and earlier [26,27,28,29] findings of a similar relationship between food intake and body weight increase in male SD rats with hyperglycaemia, and is correlated with the evident muscle mass loss. Since the rats’ motility was not visibly impaired, the BWt lowering may have been due to hyperglycaemia-induced dehydration, as indicated by increasing haemoconcentration that progressed with the duration of the diabetes; this was also reported by others [30,31].

We found that a number of physiological variables varied distinctly with the development of diabetes. Unexpectedly, in the DM-60 group, plasma sodium (PNa) appeared to be considerably lower than that in the DM-14 group. On the other hand, our diabetic animals showed elevated Posm, both in early and established diabetes. Apparently, in the DM-60 group, hyponatremia was not dilution-dependent; this does not support the view that under marked hyperglycaemia, an increase in Posm drives water out of cells, leading to hyponatremia [32], nor does this explain our finding of a decrease in plasma potassium, as shown in both DM-14 and DM-60 rats. In addition, the increase in Hct shown during short- or long-term diabetes also speaks against plasma dilution. Interestingly, haemoconcentration occurred despite the animals’ free access to water. Overall, the association of enhanced haematocrit with lowered plasma potassium concentration may suggest that sustained hyperglycaemia caused a decrease in rats’ vasopressin levels [33].

#### Duration of Hyperglycaemia and Renal Excretion

We aimed to establish if alterations in the renal function observed in conscious rats with streptozotocin-induced diabetes depended on the duration of hyperglycaemia.

In DM-60 rats, the renal excretion parameters (V and UosmV) were distinctly lowered, and this was associated with reduced water intake. Interestingly, a lower PNa in these rats (as discussed above) was not associated with any difference in sodium excretion. It should be pointed out that the effect of hyperglycaemia on the regulation of body fluid volume may differ, depending on its duration.

Taken together, in our outbred Sprague–Dawley rats, the six weeks’ difference in the duration of hyperglycaemia significantly altered several metabolic and renal function parameters. Therefore, the duration of the disease should always be taken into account in analysis and interpretation of the experimental data.

### 3.2. Blockade of Endogenous Ado and Renal Circulation

The effects of the Ado system blockade on the blood circulation in kidneys of NG and DM rats (Figure 2) indicated ADA-induced activity changes in P1R (A1R, A2R, A3R). In general, the impact of the endogenous Ado system on renal perfusion and RVR appeared to be as a vasoconstrictor in NG, and as a vasodilator in DM rats; the minor difference shown in 14-day rats became more distinct in 60-day animals. A similar pattern in the response to ADA was shown for CBF.

Taken together, our results indicate some functional prevalence of A1R over A2R activity in the renal cortex of NG, and a reversed situation could exist; again, the difference seems to be more distinct in 60-day rats. It cannot be ruled out that the reason for this difference was a higher A1R/A2R density ratio in NG compared to DM rats, which could be augmented with the age and the duration of the hyperglycaemia. However, relevant data from the literature do not conclusively support this view; although the density of Ado receptors changed in animals with prolonged hyperglycaemia, the results were often discrepant [1,2,11].

Our data of the previous study with theophylline (Theo), an A1/A2 nonselective antagonist, also indicated a similar difference in the cortical A1R/A2R density ratio between normo- and hyperglycaemic rats [21].

Ibarrola et al. (1991) suggested that under baseline conditions, endogenous adenosine is unlikely to control baseline renal haemodynamics in rats [34]. Similarly, our results suggest that the superficial cortical microvessels of the kidney are not more sensitive to adenosine compared to the deep microvessels. On the other hand, our findings in long-term hyperglycaemia show that in pathological conditions, endogenous adenosine may have a significant influence on renal haemodynamics. Noteworthily, in DM-14 rats, the described between-group difference in the response of renal perfusion (mainly cortical) to Ado inhibition was not present in the perfusion of the medulla: OMBF decreased slightly but significantly in non-diabetic rats, while IMBF was unaffected. This is compatible with the earlier evidence indicating that the Ado system’s impact on the cortical and medullary circulation could be different [21,35,36].

Nevertheless, in the 60-day rats, the abovementioned between-group (NG vs. DM) difference in the effects of an Ado blockade on cortical perfusion was also seen for circulation in the medulla: OMBF and IMBF tended to increase in NG rats, and to decrease in DM rats. Thus, unlike the short-term, in long-term hyperglycaemia animals, the renal Ado system seems to contribute similarly to the control of the cortical and medullary vasculature by causing vasodilation.

### 3.3. Endogenous Ado Blockade and Renal Excretion

Interestingly, in the 14-day group, the excretory responses of V, UosmV, and UNaV to the endogenous Ado blockade with ADA did not vary between normo- and hyperglycaemic rats, even though the parallel alteration in haemodynamics was not quite similar. In the 60-day group, the opposite changes in renal perfusion (RBF, CBF) in NG and DM were associated with similar decreases in V and UosmV (Figure 3). It cannot be excluded that in the DM-60 animals, the associated decrease in medullary perfusion (both OMBF and IMBF) contributed to an increase in tubular sodium reabsorption. On the other hand, a UKV increase in the DM-14 group occurred without any distinct changes in renal haemodynamics. This indicates that there was some alteration in tubular potassium transport, possibly mediated by an increase in aldosterone synthesis and its action on distal tubular potassium secretion. The ADA-induced reduction in adenosine and its inhibitory action on renin release may stimulate aldosterone secretion [37,38]. However, Patinha et al. (2014) found no evidence for a direct regulation of the renin–angiotensin–aldosterone system (RAAS) by adenosine receptor activation in diabetes [39].

In general, these observations favour the view that in non-diabetic and diabetic animals, alterations in the excretory function of the kidney are not simply the consequence of blood perfusion changes, but are also due to those in tubular reabsorption. It could be concluded that in older rats under basal conditions, the tubular transport of water and total solutes is, regardless of the level of glycaemia, tonically inhibited by P1R. On the other hand, their action on the tubular reabsorption of sodium and potassium was, indeed, modified by hyperglycaemia.

To summarise, the present results support the earlier information that the Ado system contributes to renal excretion also by affecting tubular transport, but this effect is only slightly improved by STZ-induced hyperglycaemia, and depends on its duration.

### 3.4. Blockade of A2a Receptors and Renal Circulation

The renal perfusion response to the A2aR inhibition in both NG and DM animals (Figure 4) helps clarify the hypothetical A2aR-dependent alterations and the net effect of these receptors’ inhibition. It is visible that in non-diabetic and diabetic rats, the tonic influence of the A2aR system on RBF and RVR was different: there was almost no effect in NG and vasodilation in DM rats, where there was no clear difference between 14- and 60-days’ hyperglycaemia. CSC did not alter CBF, which suggests A2a contributes little to the control of blood flow in the upper cortex. It seems that in diabetic rats, the prevailing A2aR controlled deep cortex circulation, and the action was not modified by the duration of the hyperglycaemia. The current data agree well with our previous study of the effects of Theo, the A1R/A2R nonselective antagonist [21].

It is noteworthy that the reported between-group disparity (NG vs. DM) in the response of renal cortical blood flow (CBF) to A2aR inhibition was not clearly visible in the medullary circulation: OMBF decreased in DM-14 rats, whereas IMBF decreased in the DM-60 group. This is in agreement with the evidence that the impact of A2aR on cortical and medullary circulation may be different [35,36,40].

Apparently, A2aR’s contribution to the control of renal cortical perfusion is augmented by hyperglycaemia, irrespective of its duration. Within the medulla, A2aR activity seems to improve circulation, either in the outer medulla (short-term diabetes) or in the inner medulla (long-term diabetes).

Additionally, no consistent or meaningful effects of the nonselective or selective antagonism of P1R receptors on MABP suggest that under the conditions of our experiments, that basal contribution of the Ado system to the state of the peripheral vasculature (TPVR) was negligible, if at all.

### 3.5. Blockade of A2a Receptors and Renal Excretion

Only in the younger rats was the renal excretion response to A2aR inhibition roughly parallel to that shown in blood circulation in the kidney. Thus, the RBF and OMBF decrease seen in DM rats was associated with an expected decrease in renal excretion; meanwhile, in NG rats, no consistent perfusion and excretion changes were seen. Interestingly, a decrease in cortical and medullary perfusion in DM-60 animals was followed by an increase in UKV, whereas in NG-60 rats, the A2aR antagonist increased UNaV without distinct alteration to renal circulation (compare Figure 4 and Figure 5). Evidently, irrespective of rat glycaemia status (NG or DM), the excretory response did not simply follow the changes in renal perfusion, but also reflected alterations in transport along the renal tubule.

It could be suggested that under basal conditions in younger rats, A2aR tonic stimulatory action on tubular water and ion transport depends on glycaemia; in contrast, in older rats, A2aR does not affect tubular water transport, while they tonically improve tubular sodium and potassium transport both in NG and DM. Thus, it seems reasonable to conclude that short-term hyperglycaemia improves A2aR contributions to tubular transport, whereas long-term hyperglycaemia abolishes them.

Regarding younger animals, this suggestion is in agreement with the established role of Ado receptors, especially of the A2aR species, as inhibitors of sodium transport beyond the proximal tubule [35,36]. It was also described that A2a may stimulate K^+^ channels along the nephron, causing potassium reabsorption or elimination in the urine [41].

The data available do not allow for a more detailed analysis of the difference in renal excretion between younger and older groups of rats. Taken together, our data add to the evidence that A2aR determines renal excretion also by changing renal tubular transport; however, this influence is modified more profoundly by short-term streptozotocin-induced diabetes.

### 3.6. Tissue NO and H_2_O_2_ and the Effects of Endogenous Ado System

In younger rats, the usual fluctuations in kidney tissue NO availability were not modified by endogenous an Ado blockade, irrespective of the level of glycaemia; however, in older rats, the dependence on glycaemia was indeed seen (Figure 6a,b). Unexpectedly, in older rats, tissue NO increased progressively in all groups (except in ADA-infused diabetic rats). This increase was concurrent with the decrease observed in the outer and inner medulla perfusion in DM-60 animals. This indicates that the role of the intact Ado system in the control of renal tissue NO was marginal, but it became pronounced with long-lasting hyperglycaemia.

However, we found recently that the Ado receptor blockade with Theo increased kidney tissue NO, both in DM-14 and NG-14 rats (with unchanged endogenous NO synthesis) [21]. The reason for this discrepancy is not clear; however, the effects of Theo may be related mainly to the antagonism of adenosine-dependent A1R and A2aR, whereas ADA could also affect A2bR and A3R. It was shown that A2bR could activate NO synthesis [42]. Currently, information about the role of A3R in diabetes is very limited.

Similarly to the Ado blockade with ADA in normoglycaemia, the A2a blockade with CSC did not affect renal tissue NO availability. Unlike with ADA, tissue NO was decreased by CSC only in the short-lasting hyperglycaemia; this was concurrent with a decrease in OMBF. While in established hyperglycaemia tissue, NO did not alter; a decrease in inner medullary perfusion was also seen. This indicates that A2aR activity appreciably contributed to the control of tissue NO bioavailability and the tone of medullary vessels, be it in the initial phase of hyperglycaemia only. In established diabetes, A2aR’s impact on the medullary vasculature was probably not mediated by NO.

Since tissue H_2_O_2_ was found to be altered by ADA solvent infusion, the data do not allow for reliable interpretation. Anyway, the role of the Ado system in control of tissue H_2_O_2_ appeared negligible, was not modified by age, and was not altered by hyperglycaemia, irrespective of its duration.

The A2R blockade with CSC altered renal tissue H_2_O_2_ in NG rats only. Interestingly, we found a decrease in tissue H_2_O_2_ after ADA, but an increase after CSC (compare Figure 6c,d and Figure 7c,d). Cowley’s group reported recently that increasing renal medullary H_2_O_2_ caused a decrease in MBF and sodium excretion in rats. These results indicate that H_2_O_2_ is a vasoconstrictor in the renal medullary vasculature, and is possibly responsible for medullary injury associated with oxidative stress [43,44]. Interestingly, in our study of NG-14 rats, changes in tissue H_2_O_2_ levels and renal medullary perfusion were concurrent, albeit in opposite directions, depending on the drug used: after ADA infusion, both medullary perfusion and tissue H_2_O_2_ decreased, whereas after CSC infusion, both medullary perfusion and tissue H_2_O_2_ increased. No such consistent changes were shown in NG-60 rats. In addition, it was noted in the NG-14 group, irrespective of whether nonselective or selective P1R activity inhibitor was used, a similar tendency to increase in UNaV was observed, while in NG-60 rats, UNaV was elevated only following the antagonist selective for A2aR. Thus, it seems that under conditions of normoglycaemia, the contributions of particular Ado receptors to tissue H_2_O_2_ bioavailability may change with the animal’s age.

On the whole, the role of P1R in the control of tissue NO in normoglycaemia appeared minor, if any, and unrelated to the age of the rats. In diabetic animals, the participation of A2aR was demonstrable only in the early phase of hyperglycaemia, whereas other P1R seemed involved also in the late phase.

On the other hand, our data indicate that the Ado system improved renal H_2_O_2_ bioavailability in normoglycaemia, but not in diabetes, regardless of its duration. The effects of CSC infusion indicate that the tonic activity of A2aR (but not of other receptor subtypes) may be responsible for the diminution of tissue H_2_O_2_ (compare Figure 6c,d and Figure 7c,d).

It should be taken into account that the necessity to apply, in studies with living animals, DMSO (dimethyl sulfoxide) as a CSC solvent, complicates the interpretation of the relevant data, even though we tried to reduce the agent’s concentration. DMSO was reported as a substance with properties to either dilate or constrict the vessels, depending on which vascular bed was actually under examination [45,46]. It should yet be noted that 10% DMSO (used as CSC solvent) did not increase the endogenous adenosine concentrations, and it did not cause any change in crucial cardiovascular parameters [47].

### 3.7. Limitations of the Study and Future Research Targets

Although Sprague–Dawley and Wistar rats are very often used to induce STZ diabetes, some concerns should be mentioned; these were described earlier in several reviews, and were recently summarised by Ghasemi and Jeddi (2023) [48]. During the first 24 h after injection of a high STZ dose, fluctuations in glycaemia were observed, eventually followed by stable hypoglycaemia. Such fluctuations are due to changing serum insulin levels, and may be responsible for post-STZ mortality. With an STZ dosage similar to ours, the mortality in Wistar rats was 25%. In male Sprague–Dawley rats, the post-STZ mortality rate (over 6 weeks) was 12%. In our study, the mortality was around 1%, and mostly occurred 5 weeks after STZ treatment.

Following recommendations provided by Ghasemi and Jeddi [48], we tried to overcome the limitations of the STZ model using the protocol described in Section 4, Materials and Methods. To avoid early post-STZ hypoglycaemia-related mortality, our rats were fasted only three hours before STZ injection and had free access to food and drinking water soon thereafter. In addition, STZ was dissolved in acidic (pH 4.5) citrate buffer to improve the drug’s stability [48]. Moreover, it was shown that rodents with glycaemia between 290–540 mg/dL can be maintained without insulin injection, but those with higher than 630 mg/dL need insulin therapy [48].

The present study has some pretty obvious limitations in the design, the model used, and some aspects of the performance. The mechanisms underlying the renal responses to exogenous ADA (deamination of adenosine) and to CSC (A2aR antagonist) are complex. We admit that the effects on blood vessel tone and on renal tubules could be different from those observed in the current research if the active agents were delivered to the renal interstitium rather than to the vascular lumen. However, earlier evidence indicates that the endogenous agonist (adenosine) acts on A2aR located in the renal vascular endothelium [7]. It would also be interesting to investigate how the effects of the A2aR blockade would compare with the antagonism of A2bR, given that both receptors act via the same cellular mechanisms [7].

### 3.8. Concluding Remarks

#### 3.8.1. The Duration of Diabetes Alters the Impact of P1R on Renal Perfusion

− short-lasting diabetes (2 weeks, DM-14) can slightly modify the joint effect of all P1R on renal blood perfusion; however, this modification differs in the cortical and medullary regions. In the cortex, the vasoconstrictor effect prevails in normoglycaemic rats, but an inverse pattern is observed in hyperglycaemia. In general, the vasoconstrictor effect of P1R is most pronounced in the medulla of diabetic animals. However, in DM rats, the vasodilator impact of A2aR does not differ between the cortex and medulla.
− long-lasting diabetes (8 weeks, DM-60) distinctly modifies the joint effect of all P1R on renal perfusion, but with no crucial difference between cortical and medullary circulation. In both regions, a constrictor effect (most probably A1R-dependent) prevails over dilatory action (most probably A2R dependent) in normoglycaemia, but this pattern is reversed in long-term diabetes.

#### 3.8.2. The Duration of Diabetes Can Also Alter the Impact of P1R on Renal Excretion

− short-lasting diabetes does not alter the net effect of all P1R on renal excretion, but can modify the impact of particular subtypes, such as A2aR.
− long-lasting diabetes blunts the joint impact of all P1R on urine sodium excretion, and also its concentration; however, it does not alter A2aR contributions to the control of renal excretory functions.

#### 3.8.3. The Duration of Diabetes Can Alter the Contribution of Individual or All P1R in Renal Tissue NO and H_2_O_2_ Availability 

Independently of its duration, diabetes exerts tonic stimulatory action of A2aR on NO bioavailability, but reduces P1R involvement in the control of tissue H_2_O_2_ shown under normoglycaemia.

## 4. Materials and Methods

The experimental procedures (study design) were mostly described in our previous studies [21,25]. The main exception was that chronic observation of hyperglycaemic animals was expanded and conducted for eight weeks after diabetes induction, before the acute experiments with ADA or CSC were performed (see Section 4.2 and Section 4.3).

### 4.1. Animals

The experimental procedures were approved by the First Local Ethical Committee, Warsaw, Poland, and were in accordance with the National Institutes of Health Guide for the Care and Use of Laboratory Animals and the European Union Directive 63/2010.

Male Sprague–Dawley rats (Tac:Cmd:SD), aged 6–7 weeks, with free access to tap water and a standard dry pellet rat diet (0.25% Na *w*/*w*, SSINFF GmbH, Soest, Germany), were housed in groups of 2–4 animals in cages with environmental enrichment, under a 12:12 h light–dark cycle and a temperature of 22–23 °C.

To improve the animals’ welfare and handling, wood scraps and an increased amount of woodchips (ca. 30%) were placed in the cages for each exchange procedure, to prevent foot skin problems during long-term observations in diabetic rats.

### 4.2. Induction of Diabetes

On day 0, after 3 hours of food deprivation, the rats were injected with streptozotocin (STZ *i.p.*; 60 mg/kg, Santa Cruz Biotechnology, Dallas, TX, USA), which was dissolved in citrate buffer (0.05 mol/L, pH 4.5) shortly before injection, in order to induce diabetes (DM), or with an equivalent volume of solvent (normoglycaemic control rats, NG). The animals were considered diabetic if, 72 h after STZ injection, their blood glucose levels were above 300 mg/dL and remained elevated until the acute experiment. Thereafter, until the acute experiment, the blood glucose level was periodically determined. This allowed for the exclusion of animals with spontaneous recovery from hyperglycaemia. The animals were randomly divided into two main experimental groups: short- (14 days) and long-term (60 days) chronic observations.

### 4.3. Chronic Studies

The day before, and on days 3, 7, and 10 (in short-term protocol), or weekly (in long-term protocol) after STZ or buffer administration, the animals were kept for 24 h in individual metabolic cages (Tecniplast S.p.A., Buguggiate, Italy) for feeding and metabolism control. The last metabolic cage observations (“Final”) were performed 3 days before the acute experiment, to allow the animals to recover from metabolic cage stress. For the sake of simplicity, we labeled them 14 and 60 days, for short- and long-term protocols, respectively. The rats’ Bwt, food and water intake, and urine volume were monitored. The Uosm, UNa, and UK were measured in urine to determine the UosmV, UNaV, and UKV. Following 2 hours of food deprivation, samples of blood were drawn from the tail vein. The blood glucose levels, Hct and Posm, PNa, and plasma K^+^ (PK) were determined. To reduce stress and obtain more reliable results, we also refined the rat immobilisation technique needed to puncture the tail blood vessels. Specifically, the rats were immobilised in plexiglass restraining tubes that were placed in warming chambers (32 °C) for 10–15 min; this helped to dilate the veins and make them clearly visible, which shortened the sampling time.

### 4.4. Acute Experiments

#### 4.4.1. Surgical Preparations

The animals were anaesthetised with intraperitoneal sodium thiopental (100 mg/kg, Valeant, Czech Pharma s.r.o.). During the surgical preparation and experimental procedures, the animals were placed on a heated surgery table, and the rectal temperature was maintained at about 37 °C. A polyethylene tracheal cannula was used to ensure free airways. The jugular vein was cannulated for fluid infusion (started with 3% bovine serum albumin solution; 10 mL/kg/h), while the carotid artery was cannulated for mean arterial blood pressure measurement (Stoelting blood pressure meter and transducers, Wood Dale, IL, USA). The left kidney was exposed from a subcostal flank incision and placed in a plastic holder, similar to that used for micropuncture. The ureter was cannulated for timed urine collection (the volume determined gravimetrically) to measure urine flow (V) and to determine the Uosm, UosmV, UNaV, and UKV [49]. A catheter was inserted through the aorta into the renal artery for the administration of drugs or the vehicles directly into the kidney; the infusion was started immediately (Ringer’s solution, Fresenius Kabi, Poland; 1 mL/h).

The total kidney blood flow (RBF) was measured using an ultrasound non-cannulating probe, 1 mm in diameter (flowmeter type T106; Transonic System Inc., Ithaca, NY, USA), and placed on the renal artery. The laser-Doppler probes of type PF 407 and two needle probes (PF 402) were placed on the kidney surface, or inserted in the kidney to different depths for measurement of blood flow through the superficial cortex (CBF) and outer (OMBF) and inner medulla (IMBF), respectively. The probes were connected with a Periflux 5010 flowmeter (Perimed, Jarfalla, Sweden).

For the amperometric detection of NO and H_2_O_2,_ signal changes in the renal tissue, needle-shaped sensors held in micromanipulators were inserted into the kidney medulla; the sensor used was either an ISO-NOP 200 sensor (0.2 mm in diameter) to the depth of 5–7 mm, or an ISO-HPO 100 sensor (0.1 mm in diameter) to the depth of 7 mm from kidney surface. Both sensors were connected with a Free Radical Analyser (TBR4100, World Precision Instruments, Sarasota, FL, USA).

#### 4.4.2. Experimental Protocol

After completing surgical preparations and placement of the renal probes, *i.v.* infusion of bovine serum albumin was replaced by Ringer’s solution at the same infusion rate. Simultaneously, an infusion of Ringer’s solution into the renal artery (*i.a.*) was started at 1 mL/h.

After 45–60 min of recovery, urine collection was conducted (four periods, 15-min each) to establish baseline diuresis and excretion of total solute, and ions of sodium and potassium. Thereafter, *i.a.* saline infusion was replaced by one of the following drugs: (i) adenosine deaminase (enzyme catalyzing irreversible deamination of adenosine to inosine; ADA, 140 units/kg; Worthington Biochemical Corp., Lakewood, NJ, USA) or Ringer’s solution (Rs) for 30 min; (ii) highly selective A2a receptor antagonist—8-(3-chlorostyryl)caffeine (CSC, 1.7 µmol/kg/h; Cayman Chemical, Ann Arbor, MI, USA) or its solvent: 8% DMSO (S, dimethyl sulfoxide; Sigma, Gdynia, Poland, diluted in phosphate buffered saline), for 60 min. Subsequently, the drug was replaced by saline for recovery periods lasting 60 min. After completing the urine collection, L-NAME, a nonselective NOS inhibitor (N^G^-nitro-L-arginine methyl ester; Sigma, Poland) was administered *i.v*. at 2.4 mg/kg, dissolved in 0.5 mL of saline. This allowed for estimating the minimal NO current, which was taken as the tissue NO signal zero level [50]. At the end of the experiment, the rats were killed with an overdose of sodium thiopental. The position of the intrarenal probes was checked at the kidney’s cross-section.

The following protocol was used in short- and long-term experimental groups (*N* = 8–10 in each):

1. normoglycaemic rats + ADA (NG + ADA)
2. diabetic rats + ADA (DM + ADA)
3. normoglycaemic rats + Ringer’s solution (NG + Rs)
4. diabetic rats + Ringer’s solution (DM + Rs)
5. normoglycaemic rats + CSC (NG + CSC)
6. diabetic rats + CSC (DM + CSC)
7. normoglycaemic rats + DMSO (NG + DMSO)
8. diabetic rats + DMSO (DM + DMSO)

#### 4.4.3. Analytical Procedures and Calculations

Blood glucose was measured with a glucometer (ACCU-CHECK Active, Model GC, Roche, Mannheim, Germany). The Posm and Uosm were measured with a cryoscopic osmometer (Osmomat 030, Gonotec, Berlin, Germany), whereas the PNa, PK, UNa, and UK were measured via a flame photometer (Flame Photometers, BWB Technologies, Newbury, UK). The excretion parameters V, UosmV, UNaV, and UKV, were calculated from the usual formulas and standardised to g kidney weight (U×V/g KWt).

To verify the in vitro responsiveness of the NO sensor, a curve relating the readings (nanoampers, nA) to known increasing concentrations of NO released from S-nitroso-N-acetyl-d,l penicillamine (SNAP) was established, as described by Zhang and Broderick [51]. This allowed for the expression of results from the in vivo studies in terms of nA, if needed.

#### 4.4.4. Statistics

The values are expressed as mean ± SEM. The data were analysed by repeated measurement ANOVA with Bonferroni’s test, in case of multiple comparisons. When two sets of data within one group or two groups were compared, two-tailed Student’s *t*-tests for paired or unpaired samples, respectively, were applied. With more than two data sets or groups, the significance of changes was evaluated with multivariable analysis of variance (ANOVA) with repeated measurements, followed by the Duncan post-hoc test. Each time before the start of analysis, the normality of the distribution was ascertained. If the assumptions for a given test were not met, appropriate non-parametric tests were used (STATISTICA, version 10.0, StatSoft. Inc. Tulsa, OK, USA). The significance border line was set at *p* = 0.05.

## Figures and Tables

**Table 1:** Baseline values measured in acute experiments before administration of the drugs tested. Mean arterial blood pressure (MABP), renal haemodynamics, and excretion were determined in normoglycaemic (NG) and hyperglycaemic (DM) male Tac:Cdm:SD rats, 14 and 60 days after vehicle or STZ injection.

	Days After Injection	NG		DM	
			*N*		*N*
MABP	14	125 ± 3	19	129 ± 3	18
(mmHg)	60	134 ± 3 ^&^	17	124 ± 3 *	19
RBF	14	4.8 ± 0.2	19	4.3 ± 0.4	18
(mL/min/g kidney weight)	60	4.8 ± 0.3	17	2.5 ± 0.2 *^&^	19
RVR	14	27 ± 2	19	35 ± 4	18
(mmHg min/mL)	60	31 ± 4	17	53 ± 5 *^&^	19
V	14	6.5 ± 0.8	17	11.1 ± 1.2 *	21
(µL/min/g kidney weight)	60	5.2 ± 0.7	15	11.6 ± 1.5 *	20
Uosm	14	858 ± 71	21	940 ± 49	21
(mosmol/kg H_2_O)	60	1019 ± 96	19	910 ± 39	20
UosmV	14	5.5 ± 0.5	19	9.8 ± 0.9 *	21
(µosmol/min/g kidney weight)	60	4.8 ± 0.4	19	10.2 ± 1.2 *	20
UNaV	14	0.6 ± 0.1	17	1.0 ± 0.2	19
(µmol/min/g kidney weight)	60	0.5 ± 0.07	18	1.0 ± 0.2 *	18
UKV	14	1.1 ± 0.1	18	0.7 ± 0.1 *	18
(µmol/min/g kidney weight)	60	0.7 ± 0.1 ^&^	14	0.8 ± 0.1	17

The streptozotocin (STZ) vehicle was citrate buffer-injected *i.p.*; NG—normoglycaemic rats (vehicle injected), DM—STZ-induced hyperglycaemic rats. RBF—whole kidney blood flow, RVR—renal vascular resistance, V, Uosm—urine flow and osmolality, respectively; UosmV, UNaV, UKV—the urine excretion of total solutes, sodium, and potassium, respectively. The values are means ± SEM; N—number of animals; *p* = 0.05 was set as borderline significance: * significantly different from the respective normoglycaemic group, ^&^ significantly different from the respective group after 14 days.

## Data Availability

All data generated or analysed during this study are included in this published article and its supplementary information files: Figure S1, S2 and Table S1.

## Supplementary Materials

The following supporting information can be downloaded at: https://www.mdpi.com/article/10.3390/ph16050732/s1, Figure S1: Effects of Ringer’s solution (Rs), adenosine deaminase solvent) on MABP, renal haemodynamics and renal vascular resistance in normoglycaemic (NG) and hyperglycaemic rats (DM) measured in acute experiments 14 or 60 days after streptozotocin or vehicle injection; Figure S2: Effects of S (solvent of A2a receptor antagonist CSC) on systemic and renal haemodynamics in normo- and hyperglycaemic rats (NG and DM, respectively) measured 14 or 60 days after streptozotocin or vehicle of streptozotocin injection. Table S1: Parameters of body weight, blood and plasma (tail vein samples, upper section) and the data of 24 h observations in meta-bolic cages (lower section) for the samples obtained before streptozotocin or solvent injections (“Initial”) and 3 days before acute experiment (“Final”) in male Tac:Cmd:SD rats untreated (NG) or pre-treated with streptozotocin (DM).
