# Supplementary material for: Nonselective and A2a-Selective Inhibition of Adenosine Receptors Modulates Renal Perfusion and Excretion Depending on the Duration of Streptozotocin-Induced Diabetes in Rats
Source: Pharmaceuticals (Basel). 2023 May 11;16(5):732. doi: 10.3390/ph16050732 (PMC10222446; doi:10.3390/ph16050732)
Supplement: Supplementary file 1 [file pharmaceuticals-16-00732-s001.zip › Figures S1 and S2.pdf]

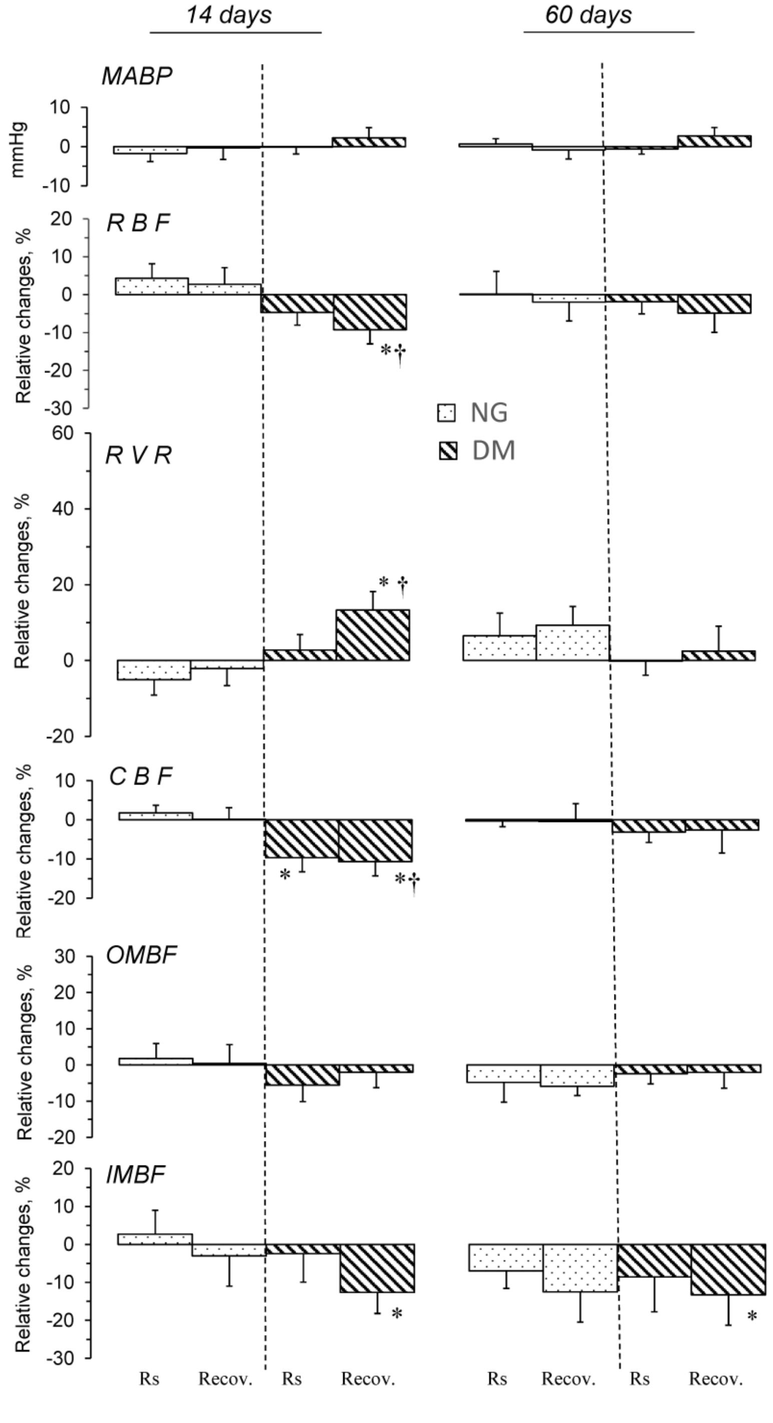


Figure S1. Effects of Ringer's solution (Rs), adenosine deaminase solvent) on MABP, renal haemodynamics and renal vascular resistance in normoglycaemic (NG) and hyperglycaemic rats (DM) measured in acute experiments 14 or 60 days after streptozotocin or vehicle injection.

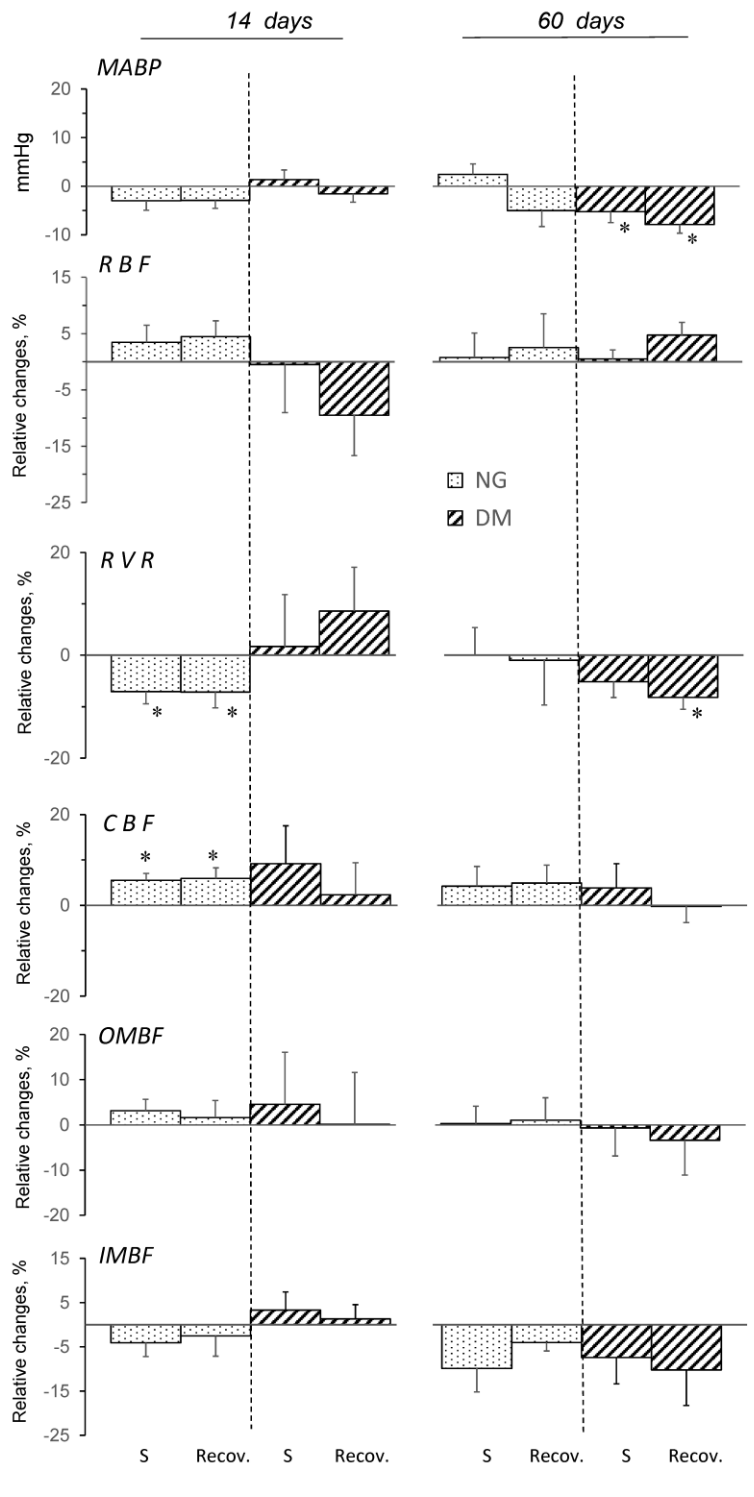


Figure S2. Effects of S (solvent of A2a receptor antagonist CSC) on systemic and renal haemodynamics in normo- and hyperglycaemic rats (NG and DM, respectively) measured 14 or 60 days after streptozotocin or vehicle of streptozotocin injection.
