# Supplementary material for: Nonselective and A2a-Selective Inhibition of Adenosine Receptors Modulates Renal Perfusion and Excretion Depending on the Duration of Streptozotocin-Induced Diabetes in Rats
Source: Pharmaceuticals (Basel). 2023 May 11;16(5):732. doi: 10.3390/ph16050732 (PMC10222446; doi:10.3390/ph16050732)
Supplement: Supplementary file 1 [file pharmaceuticals-16-00732-s001.zip › Table S1.pdf]

Table S1. Parameters of body weight, blood and plasma (tail vein samples, upper section) and the data of 24 h observations in metabolic cages (lower section) for the samples obtained before streptozotocin or solvent injections ("Initial") and 3-4 days before acute experiment ("Final") in male Tac:Cmd:SD rats untreated (NG) or pre-treated with streptozotocin (DM).

		NG			DM		
	m.c.	Initial	Final	*N*	Initial	Final	*N*
Body weight	14	232 ± 7	299 ± 3*	16	244 ± 3	256 ± 5*†	24
(g)	60	198 ± 3$^{\&}$	406 ± 7*$^{\&}$	37	204 ± 3$^{\&}$	289 ± 6*†$^{\&}$	43
Glycaemia	14	180 ± 6	163 ± 6*	16	193 ± 7	481 ± 16*†	24
(mg/dL)	60	172 ± 4	158 ± 5*	37	180 ± 3	535 ± 8*†$^{\&}$	43
Haematocrit	14	43 ± 1	43 ± 0	15	43 ± 0	46 ± 0*†	22
(%)	60	41 ± 0 $^{\&}$	46 ± 0*$^{\&}$	34	41 ± 0$^{\&}$	49 ± 0*†$^{\&}$	43
Plasma osmolality	14	297 ± 2	299 ± 2	16	299 ± 2	317 ± 2*†	24
(mosmol/kg H2O)	60	295 ± 1	306 ± 2*$^{\&}$	34	293 ± 1$^{\&}$	316 ± 2*†	43
Plasma sodium	14	134 ± 1	134 ± 1	16	132 ± 1	131 ± 1	23
(mmol/L)	60	133 ± 1	135 ± 1*	34	134 ± 1	128 ± 1*†$^{\&}$	40
Plasma potassium	14	5.7 ± 0.2	5.1 ± 0.2*	16	5.6 ± 0.2	5.1 ± 0.1*	23
(mmol/L)	60	5.6 ± 0.1	5.1 ± 0.2*	34	5.6 ± 0.2	5.4 ± 0.2†$^{\&}$	41
Food intake	14	34 ± 2	23 ± 1	10	27 ± 1	40 ± 1*†	13
(g/24 h/300 g BWt)	60	34 ± 2	17 ± 1*$^{\&}$	12	35 ± 2$^{\&}$	36 ± 3*†	12
Water intake	14	50 ± 5	33 ± 4*	9	37 ± 2†	182 ± 8*†	13
(ml/24 h/300 g BWt)	60	47 ± 2	23 ± 1*$^{\&}$	16	58 ± 8$^{\&}$	141 ± 11*†$^{\&}$	11
Urine flow	14	16 ± 2	10 ± 1*	10	13 ± 1	152 ± 6*†	13
(ml/24 h/300 g BWt)	60	17 ± 2	10 ± 1*	16	18 ± 1$^{\&}$	117 ± 9*†$^{\&}$	12
Urine osmolality	14	1956 ± 89	2156 ± 122	9	1978 ± 76	1085 ± 47*†	14
(mosmol/kg H_2O)	60	1871 ± 87	2089 ± 101*	16	1824 ± 86	1100 ± 42*†	12
Total solute excretion	14	29 ± 4	22 ± 1*	9	25 ± 2	154 ± 6*†	14
(mosmol/24 h/300 g BWt)	60	30 ± 2	20 ± 1*	16	31 ± 1$^{\&}$	125 ± 8*†$^{\&}$	12
Urine sodium excretion	14	2.5 ± 0.2	1.8 ± 0.1*	9	2.1 ± 0.2	4.1 ± 0.3*†	14
(mmol/24 h/300 g BWt)	60	2.5 ± 0.1	1.2 ± 0.1*$^{\&}$	16	2.7 ± 0.1$^{\&}$	3.9 ± 0.3*†	12
Urine potassium excretion	14	8.3 ± 0.9	5.9 ± 0.3*	9	6.6 ± 0.6	8.2 ± 0.4*†	14
(mmol/24 h/300 g BWt)	60	8.3 ± 0.5	4.2 ± 0.3*$^{\&}$	16	8.5 ± 0.4$^{\&}$	8.3 ± 0.5†	12

Streptozotocin (STZ) vehicle or STZ was injected *i.p.* NG – normoglycaemic rats (vehicle injected), DM – STZ-induced hyperglycaemic rats; m.c. - metabolic cage; Bwt - body weight.

Values are means ± SEM; *N* - number of animals; * - significantly different from the corresponding "Initial" metabolic cage; † - significantly different from the corresponding NG; & - significantly different from the corresponding 14-days value.
